# Pre-Dispersal Seed Predation in a Species-Rich Forest Community: Patterns and the Interplay with Determinants

**DOI:** 10.1371/journal.pone.0143040

**Published:** 2015-11-17

**Authors:** Yue Xu, Zehao Shen, Daoxin Li, Qinfeng Guo

**Affiliations:** 1 Key Laboratory for Earth Surface Processes of Ministry of Education, College of Urban and Environmental Sciences, Peking University, Beijing, China; 2 Dalaoling National Forest Park, Yichang, Hubei, China; 3 Eastern Forest Environmental Threat Assessment Center, United States Department of Agriculture (USDA) Forest Service, Research Triangle Park, North Carolina, United States of America; Chinese Academy of Forestry, CHINA

## Abstract

Pre-dispersal seed predation (PDSP) is commonly observed in woody plants, and recognized as a driver of seed production variability that is critical for successful regeneration. Earlier studies on PDSP and its determinants were mostly species specific, with community-level PDSP rarely estimated; and the interactions between the temporal variability of seed production and PDSP remain elusive. In this study, the community seed rain of woody plants in a mixed evergreen-deciduous broadleaf forest was monitored for seven years. We examined predation on collected seeds and analyzed the determinants of PDSP. PDSP was recorded in 17 out of 44 woody plant species, and three-quarters of PDSP was due to insect predators. Annual seed production varied substantially at community level, reversely linked with the temporal variation of PDSP rate. The PDSP rate was biased regarding fruit types, and being significantly correlated with seed mass when using phylogenetic independent contrasts (PICs) or without taking into account phylogenetic relations, especially for nuts. PDSP rate was also negatively correlated with seed density, showing a threshold-related predator satiation effect. The community-level PDSP rate was primarily determined by tree height, fruit type, and interannual variation of seed production and seed mass. Our analysis revealed a causal link between seed production and the dynamics of PDSP rate at the community level. The predator satiation effect was primarily contributed by the dominant species, whereas the rare species seemed to apply a distinct “hide-and-seek” strategy to control the risk of PDSP. The mechanistic difference of seed production between the common and rare species can shed new light on species coexistence and community assembly. Long-term monitoring of both seed rain and seed predation is required for understanding the ecological and evolutionary implications of species regeneration strategies in a species-rich forest community.

## Introduction

Seed predation has long been a central topic in population and community ecology [1, 2]. It is a critical process that takes place before and/or after dispersal and regulates the distribution and abundance of plant species. By reducing seed density around conspecific individuals [3], pre-dispersal seed predation (PDSP) can modify the species composition and spatial patterns of effective seed dispersal [4–6], therefore regulating the coexistence of plant species in the community [7]. PDSP has been commonly observed in various vegetation types [7,8], and the intensity of PDSP substantially differs among plant species due to the phenotypic characteristics of plants such as phenology [9–11], number of flowers [12,13], seed size and density [14, 15], as well as the body size of predators [16]. The ecological and evolutionary impacts of PDSP are prominent in certain ecosystems [14, 17]. For instance, PDSP by ants was found to reverse the quantity of viable seeds of two competing *Astragalus* species with different fecundity [18]. PDSP also changes the life-time species fitness via plant–predator interactions [19]. HoweverOn the other hand, plants can reduce seed predation by developing physical barriers and chemical defenses [20], and can minimize seed loss through a predator satiation effect produced by mast seeding [21, 22].

By synchronizing periodic seed production and causing a predator satiation effect, which is recognized as an evolutionary strategy in part for depressing seed predation and maximizing reproductive efficiency [23]. Although this strategy has been repeatedly tested for different plant species, its applicability for species within a community with variant population size and fecundity capacity has rarely been tested. Furthermore, it is often doubtful that the predator satiation strategy applies to species with low population density or low fecundity. Since rare species generally constitute the majority of species richness in a plant community, especially those in the tropical and subtropical humid regions [24], it is critical to understand the different responses of plant species to PDSP pressure.

Although PDSP has been long studied, most early studies were based on (1) a single year or season and (2) a single selected species or congeneric species [7], especially common species in a community. In this study, we aimed to analyze a community-level PDSP based on seven years of seed rain observations of all woody plants in a beech (*Fagus lucida*) forest. We have observed that dominant *Fagus* species as well as many common species belonging to other taxa have obviously synchronized and episodic seed production, whereas other rare species produce seeds also rarely, with little predictability. And PDSP by insects has been observed for many species. Here we addressed the following questions. (1) What is the community- and species-level PDSP in the forest community? (2) How does PDSP vary over time? (3) What are the primary drivers of the observed PDSP and how do species differ in responding to PDSP? The answers to these questions would shed new light on interspecific differences in seed production and dispersal strategy in a community and may have important implications for understanding niche differentiation and species coexistence.

## Materials and Methods

### Ethics Statement

All necessary permits were obtained for the described field studies. Our study was conducted with the permission of Dalaoling National Forest Park where the study area was located. Field studies did not involve endangered or protected species. All experiments comply with current Chinese and international laws.

### Study area

The study area is located at Dalaoling Nature Reserve (110°52'–111°01' E, 31°01'–31°08' N) in Yichang, Hubei Province of China. This region has a subtropical moist monsoon climate. According to the meteorological records nearby at 1670 m a.s.l., the annual mean temperature is 16.7°C, with the maximum and minimum monthly mean temperature of 27.3°C in July and 5.5°C in January. Mean annual precipitation is 1000–1500 mm, mostly concentrated in spring and summer [25]. The field work was carried out in a mature mixed evergreen and deciduous broadleaf forest at 1300–1495 m a.s.l. The community is dominated by *Fagus lucida*. Common woody species also includes deciduous species such as *Acanthopanax evodiaefolius*, *Dendrobenthamia japonica* var. *chinensis*, *Quercus serrala* var. *brevipetiolata* and *Sorbus folgneri* and evergreen species such as *Camellia cuspidata*, *Daphniphyllum macropodum*, *Rhododendron augustinii*, *Litsea elongata*, *Machilus ichangensis* and *Symplocos phyllocalyx* [26].

### Seed predators

Plant seeds can be attacked by a mix of insect, bird, and mammal predators. Since our study was based on seed rain collections, fruits (seeds) removed from maternal trees by mammals and birds could not be estimated very reasonably. However, predispersal seed predation is mostly carried out by small and specialized insects, many of which develop inside the seeds [26, 27]. Insects of the Three Gorges reservoir area of Yangtze River were systematically investigated, including the region where our sampling site locates. In this community, larvae of species in the families Tortricidae, Noctuidae of Lepidoptera, Curculionidae and Attelabidae of Coleoptera, Cynipidae of Hymenoptera proved to be the main pre-dispersal seed predators observed in dry fruits [28]. Several genera such as *Fagus*, *Quercus*, *Castanea* and *Carpinus* have co-occurring congeneric species of insect predators [29, 30]. For example, we found larvae of *Curculio davidi*, *Curculio dentipes* in many nuts. Damage of seeds by these larvae was due to early infection of flowers or immature fruits.

### Vegetation and seed rain sampling

We established a sampling site of 1.5 ha in the forest community in 2001, and surveyed the species composition with a unit quadrat of 20 m × 20 m. Within each quadrat, we recorded all plants by species and measured the x and y coordinates, height and diameter at breast height (DBH) of each plant with DBH > 2 cm. The exposure, slope position and slope of the quadrats were also measured [31].

In 2001, we selected 10 out of all quadrats with a stratified random sampling method for seed rain collection [32], and then randomly set up 10 seed traps in each quadrat. The traps were made of plastic gauze with an iron-wire circle frame of diameter of 1 m and area of 0.785 m^2^. All traps were set 1 m above the ground to avoid post-dispersal seed predation by mammals [26].

As seed dispersal of most trees in the *F*. *lucida* community starts in August and lasts to winter [31], we began seed collection on 1 August each year. We collected seeds every 3 days and ended collection when we had five consecutive zero harvests from all 100 traps (usually in late November or early December). The contents of the seed traps were brought back to the laboratory where we counted all seeds of each species. Since 2006, we began to check the existence of predation mark for each seed under a magnifier. Each seed was classified into five categories following Shibata *et al*. [29]: (1) sound (attaining mature seed size with a sound cotyledon and/or endosperm); (2) immature (failing to attain mature seed size and color); (3) empty (flat seed with the embryo undeveloped or lacking); (4) holed (having a hole or gnawing mark caused by insect predation); and (5) broken (mainly caused by vertebrate predators). Such visual identification of seed status is commonly used in pre-dispersal predation research [33, 34].

Biological features of the plant species were considered to explore the mechanisms for the interspecific variation in PDSP (S1 Table). The information regarding fruit type and tree height of all species in the community was collected from *Flora of China* [35]. The thousand seed weight was measured for all species based on our collections of seeds.

### Data analysis

For each sampling year, species-specific seed density was calculated as the number of seeds of each species per trap per year. PDSP rates were measured as the percentage of seeds damaged in each seed trap by seed predators (i.e., insects or vertebrates).

We explored the correlation between pre-dispersal predation rate and seed density using quantile regression, which facilitates analysis from the minimum to maximum response rather than the mean or median [36], and provides a more complete picture of the relationships between variables missed by other regression methods [37]. In the case of PDSP, the upper limit of the predation rate may be more relevant than the mean, because synchronized seed production dynamics might cause predator satiation and set limits only on the maximum value [17].

Over the last few decades, researchers have recognized that comparisons across taxa may not be statistically independent from one another, since species and higher taxa are part of a hierarchically structured phylogeny [38,39]. To account for the effect of evolutionary origin on extant trait variation, the phylogenetic independent contrasts (PICs) was developed and has been frequently applied in exploring the correlations among functional traits across a group of species [40]. For the same reason, we applied PICs here to evaluate the relationship between seed mass and PDSP rate to avoid the bias related with phylogenetic structure across species. Specifically, we created the phylogeny using the ‘Phylomatic’ software (http://phylodiversity.net/phylomatic/). To resolve polytomies, randomization was carried out with function ‘multi2di’ of the package ‘picante’. The PICs were then calculated using the ‘pic’ function of package ‘picante’ (R3.0.1, R Development Core Team).

We also compared the PDSP rates among the six fruit types and test the significance of differences using the *Kruskal–Wallis test* because of the deviation from normal distribution in the data.

Generalized linear mixed model (GLMM) with crossed random effects was used to account for the variation of species-specific predation rate with the potential predictive variables. Data on PDSP rate was analyzed with a Binomial error family. Seed mass, seed density, fruit type, tree height, population abundance (represented by basal area of trees), and time of collection (year) were used as fixed factors. Fixed effects showing large ranges of variation were standardized. The seed predation data had a hierarchical structure: individual trap (level 1) was nested within each of the quadrat (level 2), which was fitted in the random-effect term. GLMM is appropriate to analyze the seed predation data with this type of spatial structure because it can quantify the relationships between the binomial response and covariates at different scales. We also included species identity as a crossed random factor in this study, such that the species analyzed here could be regarded as being randomly sampled from all species in this community [41–44]. GLMM was fitted by the lmer() function of the “lme4” package (with the default Laplace approximation to the log-likelihood) in R. Pseudo-R square following the method proposed by Nakagawa and Schielzeth [45] was used to estimate the contribution of fixed effect variables and random effect variables. To obtain the best model, different models including different numbers of variables were constructed. Model reduction and model selection were performed based on significant differences (chi-square test) between models, and we selected the model with the lowest AIC.

## Results

### General statistics of seed production and PDSP

A total of 53,509 seeds were collected from the 100 seed traps during the 7-year study (2006–2012). The seeds were from 44 woody plant species belonging to 26 families and 36 genera. Among them, only 2285 seeds (4.27% of all seeds) of 17 species (9 families and 15 genera) were damaged by animals (Table 1). About 74.9% of the predated seeds were holed (by mainly insects) and 25.1% seeds were broken by mammals and birds.

**Table 1:** Seed rain density and PDSP rates of the 17 preyed species at the study site during 2006–2012.

Species	Species abundance	Seed density (seeds/m^2^•a)		PDSP rate (%)	
		Mean	SD	Mean	SD
*Fagus lucida*	417	17.71	43.30	6.45	11.26
*Acanthopanax evodiaefolius*	124	5.38	4.35	1.73	1.68
*Sorbus folgneri*	121	2.51	3.12	37.11	33.03
*Dendrobenthamia japonica *var. *chinensis*	114	0.18	0.36	0.38	1.01
*Carpinus viminea*	101	1.80	2.08	18.23	36.36
*Quercus serrata *var. *brevipetiolata*	81	0.89	0.88	55.64	10.00
*Bothrocaryum controversum*	37	17.04	27.08	12.19	10.33
*Cyclobalanopsis myrsinifolia*	27	0.09	0.20	8.33	14.43
*Quercus aliena *var. *acutiserrata*	26	0.03	0.05	16.19	31.65
*Acer davidii*	14	0.18	0.39	1.72	4.55
*Castanea seguinii*	10	0.15	0.15	32.86	41.01
*Fagus engleriana*	8	0.03	0.07	2.04	5.40
*Aralia chinensis*	5	19.21	37.22	0.44	1.17
*Decaisnea insignis*	5	0.34	0.91	0.23	0.60
*Swida hemsleyi*	5	1.94	4.34	17.28	29.27
*Elaeagnus *sp.	4	0.01	0.01	4.76	12.6
*Cyclocarya paliurus*	2	0.60	0.91	0.15	0.39

SD, standard deviation; population abundance was recorded in the 1.5 ha plot including all individuals with DBH > 2.0cm.

**Fig 1:**
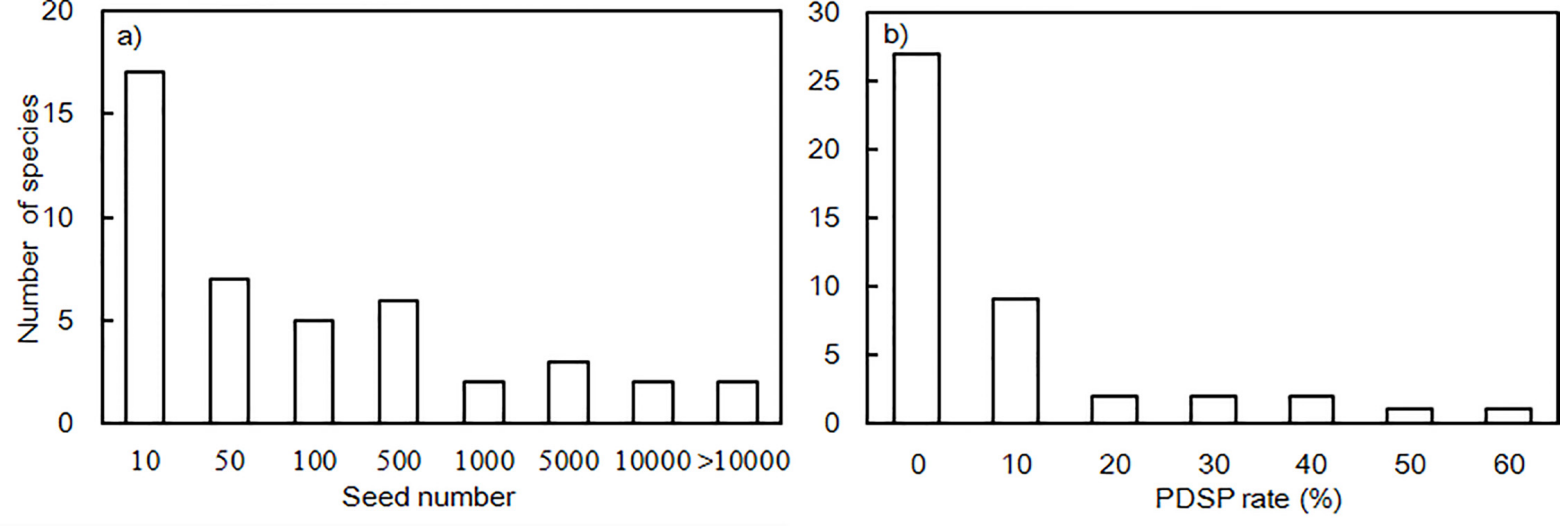
Overall seed number and PSDP rate in the seven years of study. (a) Frequency distribution of seed density of the species collected in the 100 seed traps in 2006–2012. (b) Frequency distribution of PDSP rate in 2006–2012 (total number of species = 44).

The total annual harvest of seeds in the seven years was within the range of 1–14,580 (mean ± SD of 1216.1 ± 3253.0) for all 44 species, and the PDSP rate was 0–55.6% (mean ± SD of 6.1 ± 12.8%, n = 44). Both showed positively skewed distributions (Fig 1), indicating that most plant species had very low seed production and predation rates.

The dominant species of the forest community, *F*. *lucida*, was a major source of the community seed rain, while a companion species, *B*. *contriversum*, and a rare species, *A*. *chinensis*, also made comparable contributions to the collected seeds (Table 1).

### Temporal variation of seed production and PDSP

**Fig 2:**
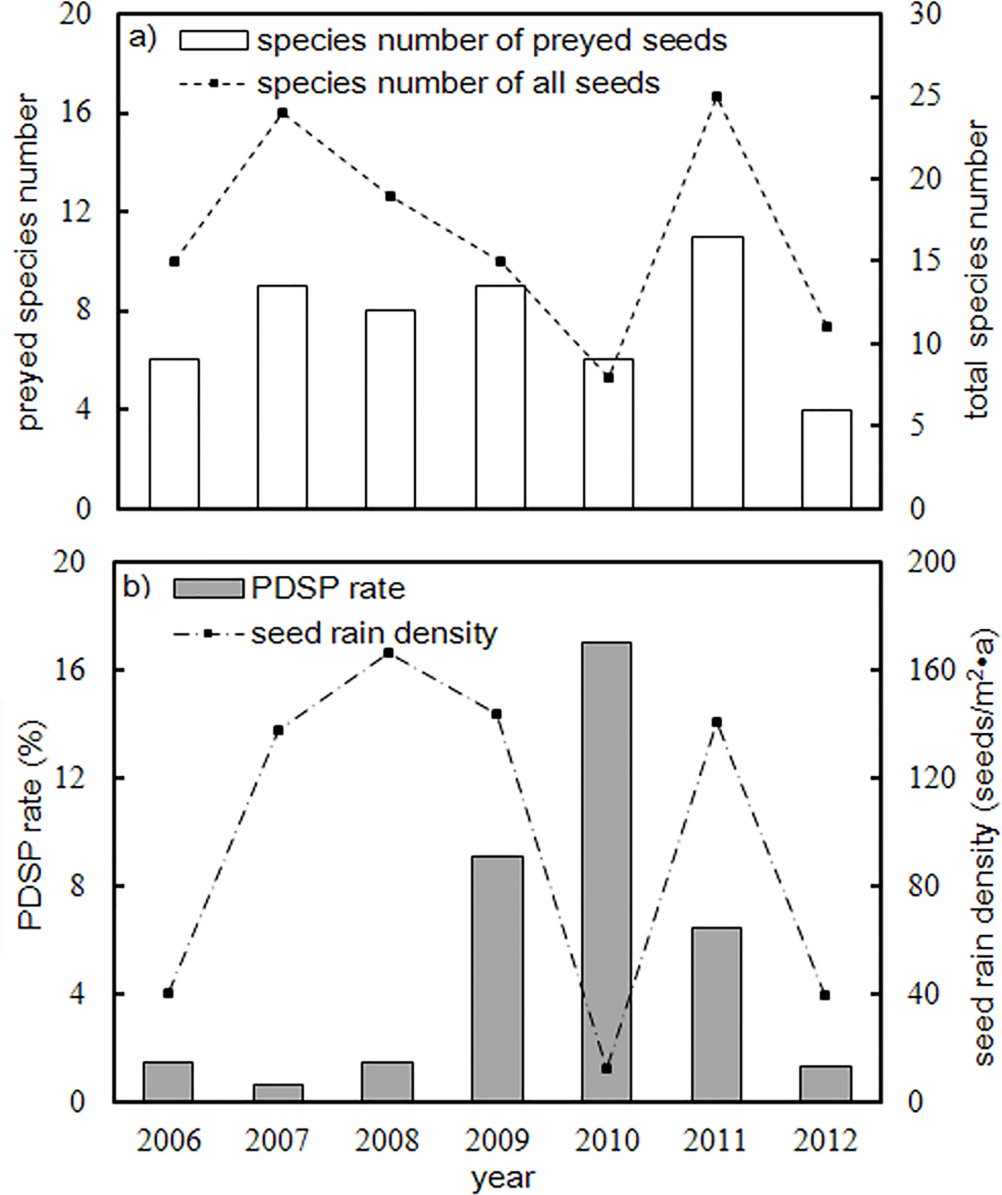
Temporal variation of seed rain and PDSP rate. (a) Interannual variations of total and preyed species richness. (b) Interannual variations of total seed rain density and PDSP rate.

Substantial interannual variations was revealed in the 7-year records of seed rain and PDSP rate (Fig 2A). Although the species richness of seed rain also fluctuated substantially in the seven years, the number of species with predated seeds was relatively stable (Fig 2B), and the species richness of predated seeds and all seeds were significantly related to each other (*R* ^*2*^ = 0.783, *p* = 0.003). However, seed rain density and PDSP rate fluctuated in opposite directions (Fig 2B). The maximum PDSP rate (17.01%) occurred after three years of high seed production in correspondence to a minimum annual seed production. Nevertheless, the fluctuation of PDSP rate was not significantly related to the dynamics of seed rain density (*R* ^*2*^ = 0.110, *p* = 0.467).

### Determinants of PDSP rate

About 22.6% of the overall variation in PDSP rate could be accounted for by the fixed and random factors included in the GLMM model (Table 2). Tree height appeared to be the primary determinant of PDSP, followed by fruit type. The effects of seed density and year were also significant. Seed mass had significant effect although with smaller contribution. However, population abundance as represented by the sum of basal area in a plot of the tree species in the community had no significant effect on seed predation.

**Table 2:** Parameter estimates and variance predictions in the generalized linear mixed model used to test for the effects of selected variables on predispersal seed predation (PDSP) at the community level.

Random effects	Variance	Fixed effects	P-value	R^2^/%
Quadrat	0.075	tree height	<0.001	5.84
trap:quadrat	0.272	fruit type	<0.001	4.92
Species	0	seed density	<0.001	4.44
		year	<0.001	3.51
		seed mass	0.018	0.74
		basal area	0.553	0.09

### Effects of seed mass and fruit type on PDSP

**Fig 3:**
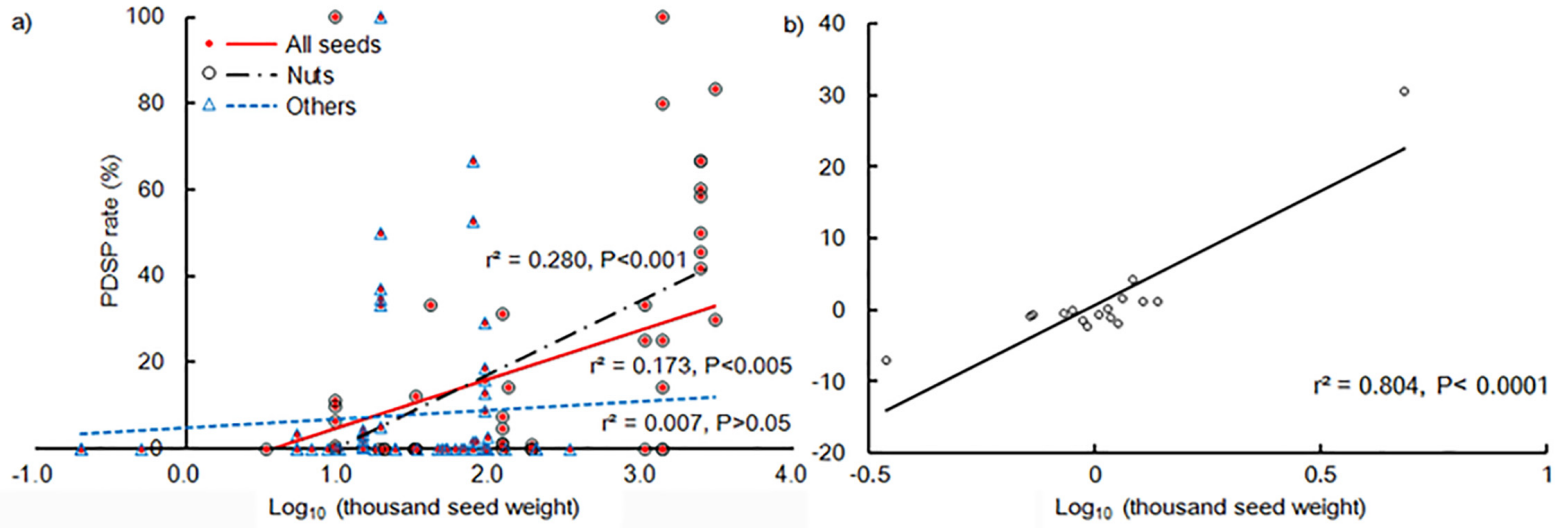
Relationship between PDSP rate and seed mass (g; log_10_thousand seed weight). (a) Pearson correlation coefficients between PDSP rate and seed mass., Different lines indicate the correlations between PSDP rate and seed mass of all species, species with nuts, and species with other fruit types. (b) Independent contrast correlations for the 17 species in the community.

The annual PDSP rate was significantly related to seed mass (*R* ^2^ = 0.173, *p* < 0.005; solid line in Fig 3). To avoid the non-independence among species, we also calculated the PICs of seed mass and pre-dispersal predation rate. The relationship between these two traits was supported by significant phylogenetically independent contrast correlations (*R* ^2^ = 0.804, *p* < 0.001). Specifically, the correlation between seed mass and the predation rate for nuts was even greater (*R* ^2^ = 0.280, *p* < 0.0001, dot dash line), whereas there was no significant correlation when all other fruit types were pooled (*p* = 0.530). Since there were only one or a few species in each fruit type, except for nuts, in the collected seeds, our data were not adequate for statistically testing the relationship between seed mass and PDSP rate for other fruit types.

**Fig 4:**
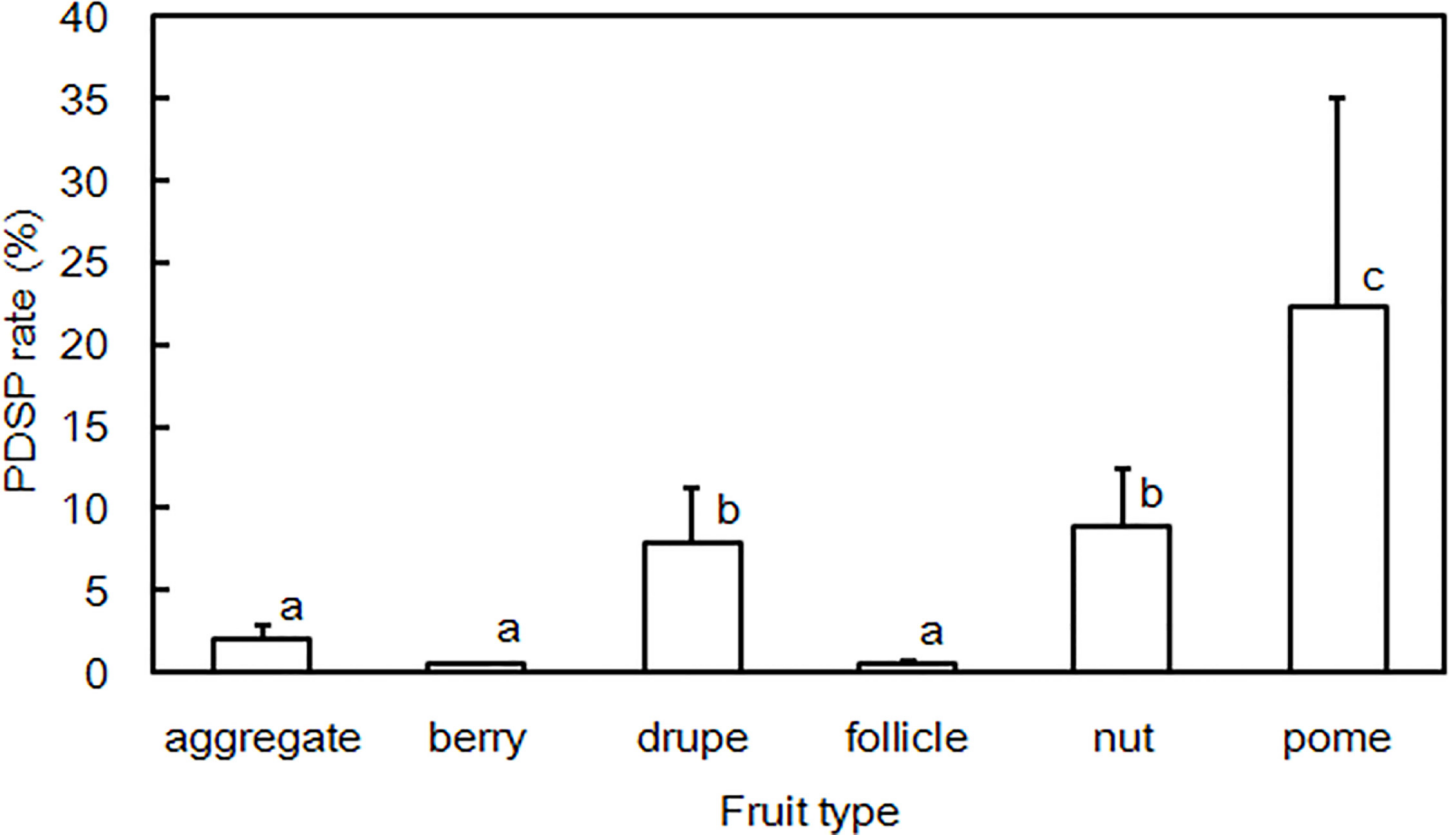
PDSP rates of six fruit types in the seven years of study. The boxes are mean PDSP rates, and the error bars indicate the standard errors. The pair-wise differences in mean predation rates between fruit types were examined with a non-parameterized *Kruskal-Wallis* test, and a, b, c represents significantly different mean values.

There were six types of fruits in our seed collection, nuts, drupes and pomes constituted the majority of the predated seeds, with 46.77, 36.87 and 13.51%, respectively. The PDSP rate substantially differed among different fruit types (Fig 4). The *Kruskal–Wallis* test showed that pomes, nuts and drupes had significantly higher mean PDSP rates than that of follicles, berries and aggregate fruits.

### Links between population, seed production and PDSP

**Fig 5:**
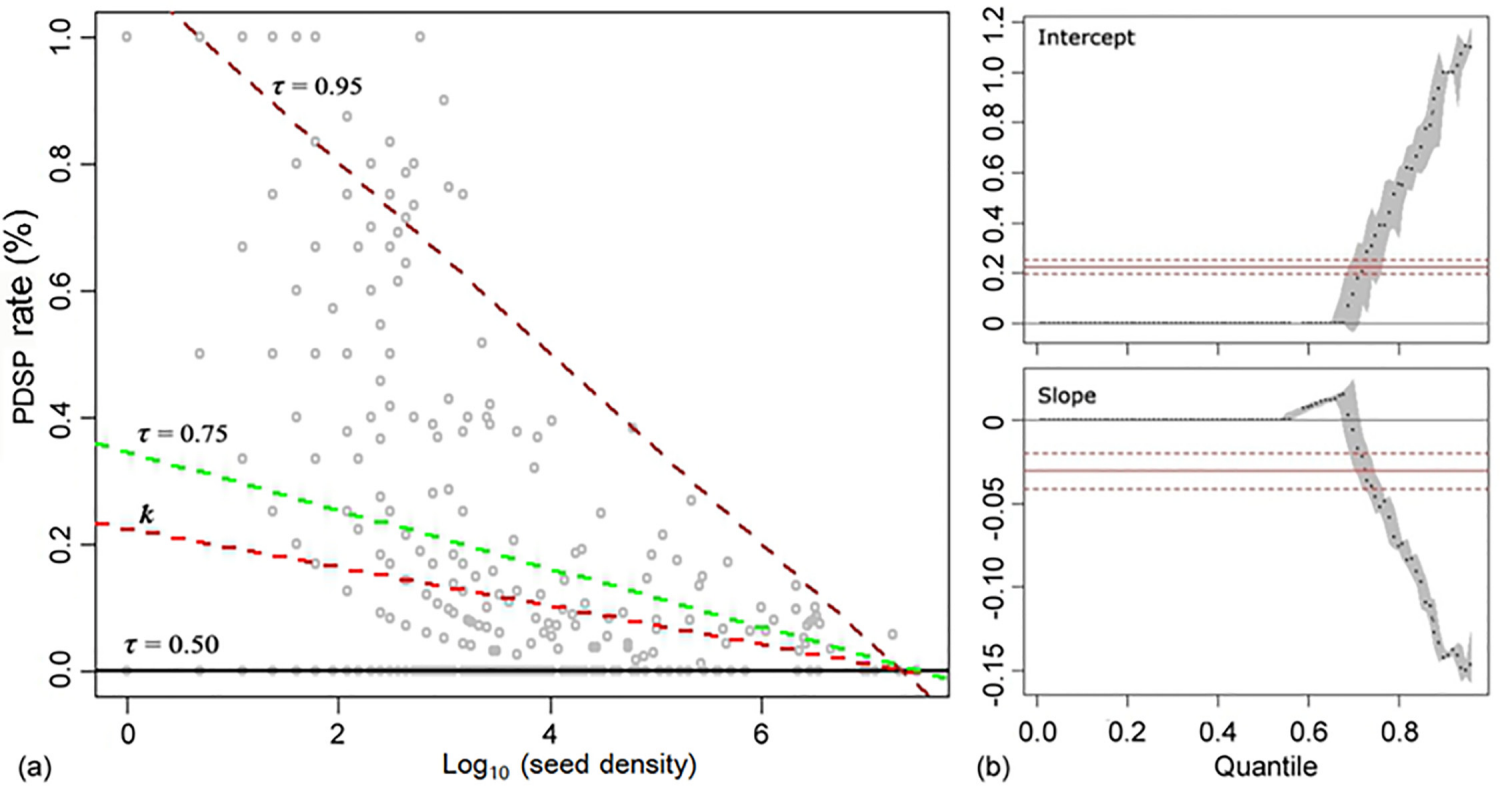
Results from quantile regression analysis between seed production (log-transformed) and PDSP rate of 17 preyed species. (a) Scatterplot of seed density and predation rates (left), with the lines of quantile (brown, τ = 0.95; green, τ = 0.75; black, τ = 0.50) and least squares regression (red line) estimates for the model Y = *β*_0_ + *β*_1_ ln(X) + ε. **(b)**. Parameter estimates, *β*_0_ (τ) (top right) and *β*_1_ (τ) (bottom right), are shown as black solid points. Grey area represents 95% confidence interval.

Although there was no significant correlation between seed production and PDSP rate (*p* > 0.05, red line in Fig 5), the predation intensity was always low for high seed rain density. The slope (*β*_1_) of the quantile regression model showed an increasing negative correlation between seed production and PDSP rate at quantiles > 0.7, indicating a threshold seed production for the occurrence of predator satiation, and the value of *β*_1_ = -0.15 set an upper limit of PDSP rate on the annual seed production.

**Fig 6:**
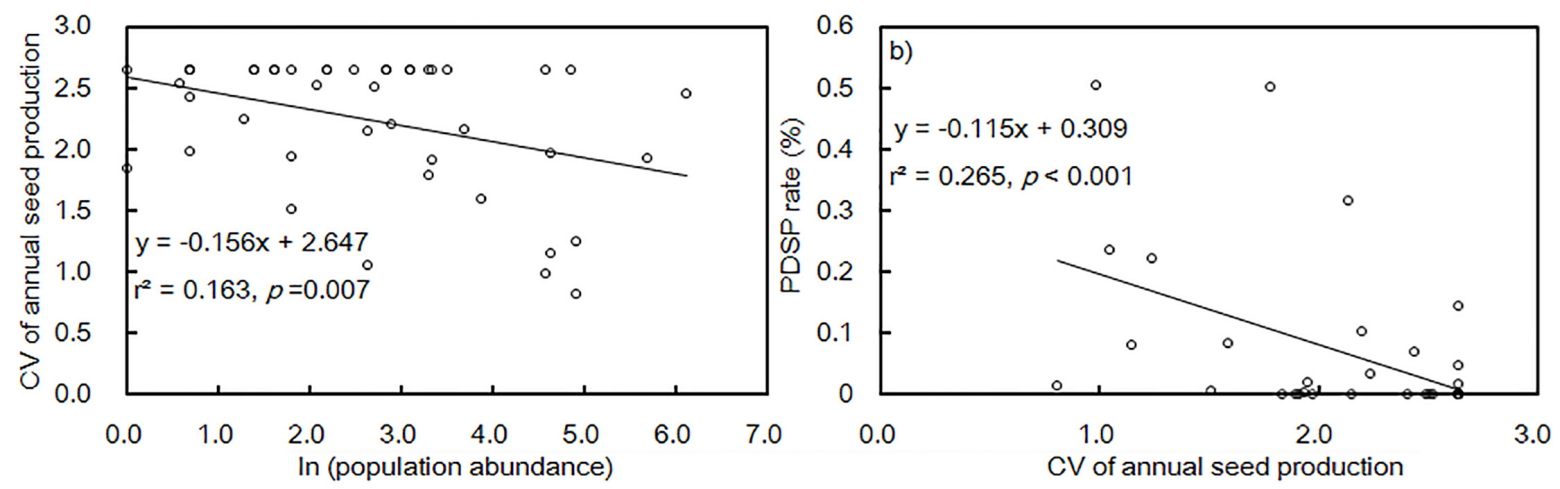
Relationship between population, seed production and PDSP rate. (a) Correlation between species specific population abundance (log-transformed) and coefficient of variance (CV) of annual seed production; (b) correlation between CV of annual seed production and PDSP rate of all woody species in the community.

The coefficient of variation (CV) of annual seed production is an indicator often used to estimate the extent of seed masting, and the CV value of sound seed was 2.23 in this community from 2006 to 2012. The species-specific population size was negatively correlated with the coefficient of variance (CV) of annual seed rain density (P = 0.007, Fig 6A). The CV of annual seed rain density was also negatively correlated with the species-specific PDSP rate (*p* = 0.001, Fig 6B).

## Discussions

### Impact of PDSP on seed production of the community

PDSP was inferred as a driver of synchronous and periodic seed production [46]. This study provides a quantitative assessment of the effect of PDSP intensity on the stock of seed production at both community and species levels. Our data showed that about 75% of the predated seeds in this study were holed because most insect predators such as weevils and moths laid eggs in immature seeds and develop larvae, especially in acorns [47].

At the community level, the PDSP rate was within the range of 0.7–17.0% in the seven years of observations, and the mean proportion of predated seeds was low (4.3%) compared with the few other community-level estimates, such as in a sclerophyllous forest in central Chile where the PDSP rate was > 20% [5].

At the species level, the PDSP varied within the range of 0–50.2% with seed mass and fruit type. Seed mass accounted for about half of the variation in seed predation, and was a primary cause of selective PDSP, as reported in earlier studies [17, 48]. PDSP was also highly selective regarding fruit type. For drupes, nuts and pomes (which constituted 62.5% of all tree species in the community, 77.3% of the species in the seed rain collection and 82.4% of the species with PDSP), the mean predation rates of these three fruit types were > 15% (e.g. 46.8% for nuts), while the impact of PDSP on other fruit types was much lower (< 3%).

### Interactive impacts of seed mass and fruit type on PDSP

Seed size (or mass) is a critical factor influencing the probability of predation in general, with larger seeds experiencing higher PDSP rates for reasons such as higher noticeability [49,50], time of exposure to predators [51], and availability for both large- and small-sized predators [27]. However, this hypothesis has not been consistently supported by empirical observations [27,52]. Predator size might be an intervening factor in this complicated relationship. Indeed, when colorful and edible pulps rather than seeds are the target of predators such as vertebrates [53,54], small seeds have a higher risk of being swallowed and dispersed undamaged, especially in long-distance seed dispersal [55].

Our data showed a significant positive relationship between seed mass and predation rate for all counted seeds (Fig 4), among which there was a much higher correlation for nuts, but no significant correlation for the other five fruit types. This result suggests that different types of predators preferred different fruit (or seed) types. Our data provide solid support for the positive correlation between seed mass and insect seed predation, although we could not adequately assess the relationship between seed mass and PDSP for other fruit and predator types due to limited data.

### Implication of seed production dynamics: more than predation satiation?

By causing predator satiation, interannual seed production variability is regarded as an adaptive strategy, in addition to other physical and chemical mechanisms, that plants evolve to reduce the predation pressure on population regeneration [1,17, 56–58]. The CV of annual sound seed over 1.5 was used to indicate the presence of seed masting [59], and in this community our estimate of CV value for sound seeds was larger than 2.0. Moreover, the variation of total seeds between years showed significant effect on the decrease of pre-dispersal seed predation (Fig 6). Our results provide community-level support to the predator satiation hypothesis, and stress that the relationship between seed production and predator satiation may not be linear but rather a threshold-related phenomenon (Fig 5). In other words, only when the seed production surpasses a threshold, would PDSP lose control of the surplus seeds, offering an opportunity for viable seed dispersal and successful regeneration. The generality of this hypothesis for species with different abundances in a community has never been addressed. Moreover, it is doubtful that most species in a community, except the dominants, have adequate seed production to cause predator satiation. A related and critical question is how a plant species minimizes seed loss due to predation when it does not have adequate fecundity capacity.

For all species in the community with seed collections, the negative correlation between population abundance and the CV of annual seed production implies that rarer species tends to be more variable in annual seed production, leading to a lower risk of PDSP (Fig 6). Although there was no direct link between population abundance and PDSP rate for all woody species, there was a significant positive correlation for the rarer species with seed predation (*R* ^*2*^ = 0.338, *p* = 0.028) when the five most abundant species were excluded.

**Fig 7:**
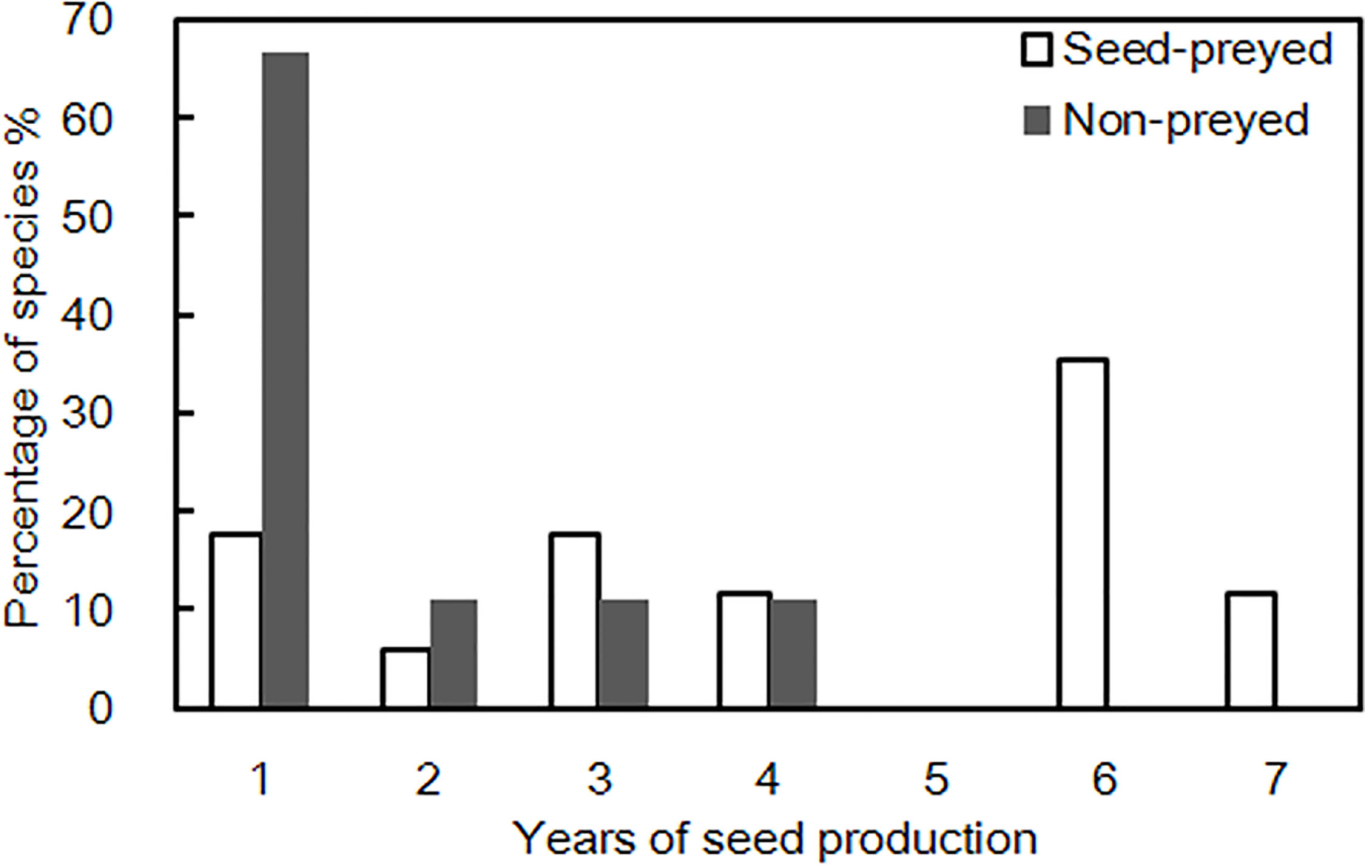
The proportion (%) of years of seed production among species with (white bar) or without (dark grey bar) seed predation.

The above results suggest that, differing from the abundant species which depress the risk of PDSP by predator satiation, rarer species in the forest community tend to rely on a distinct mechanism, i.e. reducing the PDSP risk with a larger interannual variability, therefore lower predictability in annual seed production. We refer to this as a ‘hide-and-seek’ strategy in contrast to the satiation strategy adopted by abundant species. The distinction between these two mechanisms is also revealed in the frequency distribution of seed production in our seven years of observations. For example, for 76.5% of the species with PDSP, seed production was recorded in ≥ 3 years, whereas in 77.8% of the species free of PDSP, seed production occurred in only 1–2 years (Fig 7).

### 4.3 Data uncertainty

Both mammals and birds are important pre-dispersal seed predators [14], and tend to remove fruits (seeds) from maternal trees [60]. Since most seed production studies have been based on seed rain collections, the depletion of viable seeds before dispersal is therefore easily overlooked [33, 61]. In our study, we were unable to provide a reasonable estimate of the amount of PDSP by vertebrates, and therefore our estimation of community-level PDSP was conservative in general. Moreover, since the seed production was usually estimated for all seeds rather than viable seeds [62], the magnitude of PDSP and its ecological and evolutionary effects were likely to be underestimated. Seed production variation is usually estimated to seed production of the individuals or populations at species level [58]. In the seven years seed rain monitoring, the interspecific synchronization of seed production has also been observed in the studied community (to be presented in a different paper), although did not apply to all species. Although our plot based seed collection approach (applied in most other cases) did not provide individual-based seed rain sources and seed predation, seven years of fixed monitoring can achieve a reasonable estimate of “on-site” link between the dynamics of seed production and pre-dispersal seed predation, as revealed by the effect of variable “year” in the GLMM model. However, more accurate spatially corresponding information in seed predation, insect predator, and seed production by individuals of a specific species is required to fully understand the interspecific interactions among the plant species in responding to predispersal seed predators.

## Conclusions

Relative to numerous earlier studies on species-specific PDSP, our observation of seed rain dynamics provides a novel assessment of the comprehensive dynamics of community-level PDSP and interspecific variation related to multiple functional traits. In our study, community-level PDSP is largely regulated by tree height, fruit type, seed production variation and seed mass. The temporal variability of synchronized periodic seed production seems to be related to different mechanisms for common vs. rare species, i.e. predator satiation for the former, and a ‘hide-and-seek’ strategy for the latter. These results help improve our understanding of the animal–plant interactions in plant community dynamics by filling the information gap regarding critical but often ignored pre-dispersal events.

## Supporting Information

S1 Table Life history features of the tree species in the *Fagus lucida* community at Dalaoling Nature Reserve, Hubei, China. (DOCX)
