# Supplementary material for: Pre-Dispersal Seed Predation in a Species-Rich Forest Community: Patterns and the Interplay with Determinants
Source: PLoS One. 2015 Nov 17;10(11):e0143040. doi: 10.1371/journal.pone.0143040 (PMC4648506; doi:10.1371/journal.pone.0143040)
Supplement: S1 Table — (DOCX) [file pone.0143040.s001.docx]

**Supporting Information**

**S1 Table. Life history features of the tree species in the *Fagus lucida* community at Dalaoling Nature Reserve, Hubei, China.**

| **Species** | **Family** | **Growth form** | **Fruit type** | **SM_1000_ / g** |
| --- | --- | --- | --- | --- |
| *Acanthopanax evodiaefolius* | Araliaceae | Shrub | Berry | 14.46 |
| *Acer davidii* | Aceraceae | Tree | Nut | 31.99 |
| *Aralia chinensis* | Araliaceae | Shrub | Berry | 5.30 |
| *Bothrocaryum controversum* | Cornaceae | Tree | Drupe | 91.41 |
| *Carpinus viminea* | Betulaceae | Tree | Nut | 9.43 |
| *Castanea seguinii* | Fagaceae | Tree | Nut | 1348.96 |
| *Cyclobalanopsis myrsinifolia* | Fagaceae | Tree | Nut | 1049.54 |
| *Cyclocarya paliurus* | Juglandaceae | Tree | Nut | 186.21 |
| *Decaisnea insignis* | Lardizabalaceae | Shrub | Follicle | 80.72 |
| *Dendrobenthamia japonica var. chinensis* | Cornaceae | Tree | Aggregate | 97.05 |
| *Elaeagnus*sp. | Elaeagnaceae | Shrub | Nut | 40.00 |
| *Fagus engleriana* | Fagaceae | Tree | Nut | 131.52 |
| *Fagus lucida* | Fagaceae | Tree | Nut | 119.95 |
| *Quercus aliena*var. *acutiserrata* | Fagaceae | Tree | Nut | 3026.91 |
| *Quercus serrata*var. *brevipetiolata* | Fagaceae | Tree | Nut | 2415.46 |
| *Sorbus folgneri* | Rosaceae | Tree | Pome | 19.07 |
| *Swida hemsleyi* | Cornaceae | Shrub | Drupe | 76.69 |
| *Acer amplum* | Aceraceae | Tree | Nut | 31.50 |
| *Acer flabellatum* | Aceraceae | Tree | Nut | 20.30 |
| *Acer oliverianum* subsp. *oliverianum* | Aceraceae | Tree | Nut | 19.90 |
| *Actinidia* sp. | Actinidiaceae | Liana | Berry | 0.50 |
| *Akebia trifoliata* | Lardizabalaceae | Liana | Follicle | 357.26 |
| *Camellia cuspidata* | Theaceae | Shrub | Capsule | 335.30 |
| *Carpinus cordata* var. *chinensis* | Betulaceae | Tree | Nut | 18.70 |
| *Corylopsis sinensis* | Hamamelidaceae | Shrub | Capsule | 17.30 |
| *Daphniphyllum macropodum* | Daphniphyllaceae | Shrub | Drupe | 76.40 |
| *Fraxinus insularis* | Oleaceae | Tree | Nut | 3.30 |
| *Hamamelis mollis* | Hamamelidaceae | Shrub | Capsule | 51.30 |
| *Holboellia grandiflora* | Lardizabalaceae | Liana | Follicle | 452.35 |
| *Ilex pernyi* | Aquifoliaceae | Shrub | Drupe | 9.80 |
| *Lindera erythrocarpa* | Lauraceae | Shrub | Drupe | 66.70 |
| *Lindera obtusiloba* | Lauraceae | Tree | Drupe | 59.50 |
| *Magnolia sprengeri* | Magnoliaceae | Tree | Follicle | 125.00 |
| *Pieris formosa* | Ericaceae | Shrub | Capsule | 0.20 |
| *Rhus chinensis* | Anacardiaceae | Tree | Drupe | 8.50 |
| *Sabia campanulata* subsp. *Ritchieae* | Sabiaceae | Liana | Drupe | 6.70 |
| *Sargentodoxa cuneata* | Sargentodoxaceae | Liana | Berry | 48.00 |
| *Smilax* sp. | Smilacaceae | Shrub | Berry | 65.00 |
| *Symplocos paniculata* | Symplocaceae | Shrub | Drupe | 52.00 |
| *Symplocos phyllocalyx* | Symplocaceae | Tree | Drupe | 44.70 |
| *Tilia paucicostata* | Tiliaceae | Tree | Drupe | 199.00 |
| *Toxicodendron trichocarpum* | Anacardiaceae | Tree | Drupe | 33.30 |
| *Viburnum betulifolium* var. *betulifolium* | Caprifoliaceae | Shrub | Drupe | 23.50 |
| *Zanthoxylum bungeanum* | Rutaceae | Tree | Follicle | 10.06 |

SM_1000_, weight of 1000 seeds.
